# Supplementary material for: Polymyxin B lethality requires energy-dependent outer membrane disruption
Source: Nat Microbiol. 2025 Sep 29;10(11):2919–33. doi: 10.1038/s41564-025-02133-1 (PMC12578643; doi:10.1038/s41564-025-02133-1)
Supplement: Supplementary file 1 — Supplementary Figs. 1–8 and Table 1. [file 41564_2025_2133_MOESM1_ESM.pdf]

nature microbiology

Supplementary information

https://doi.org/10.1038/s41564-025-02133-1

Polymyxin B lethality requires energy-dependent outer membrane disruption

In the format provided by the authors and unedited

Species/Strain	Relevant characteristics and source	PmB MIC (μg ml^{-1})
E. coli MG1655	Well characterised K-12 laboratory strain [92]	0.25
	P*mcr-1* [This study]	1*
	P*mcr-1** [This study]	0.25
E. coli MC4100	Well characterised K-12 laboratory strain [93]	0.25
*E. coli imp*4213	Impaired OM barrier function due to a deletion in *lptD* [94,95]	0.125
E. coli CFT073	Uropathogenic clinical isolate [96]	0.25
E. coli KPC BM16	Clinical isolate [28,97]	0.5
E. coli DIN	Clinical isolate [28,97]	0.25
E. coli ATCC 25922	Type strain used as a reference for antibiotic susceptibility testing	0.25
K. pneumoniae IMP12	Clinical isolate expressing IMP carbapenemase [98]	0.5
K. pneumoniae IMP76	Clinical isolate expressing IMP carbapenemase [98]	1
C. freundii IMP61	Clinical isolate expressing IMP carbapenemase [98]	0.5
E. asburiae IMP8	Clinical isolate expressing IMP carbapenemase [98]	1
P. aeruginosa PA14	Well characterised laboratory strain [99]	0.125
P. aeruginosa AK3	Isolate from person with cystic fibrosis. Small colony variant phenotype [27].	0.5
P. aeruginosa AK11	Isolate from person with cystic fibrosis. Mucoid phenotype [27].	0.125
A. baumannii AS	Clinical isolate [This study]	0.25
A. baumannii N12	Clinical isolate [This study]	0.25

*For MG1655 P*mcr*-1, the uninduced MIC value is provided.

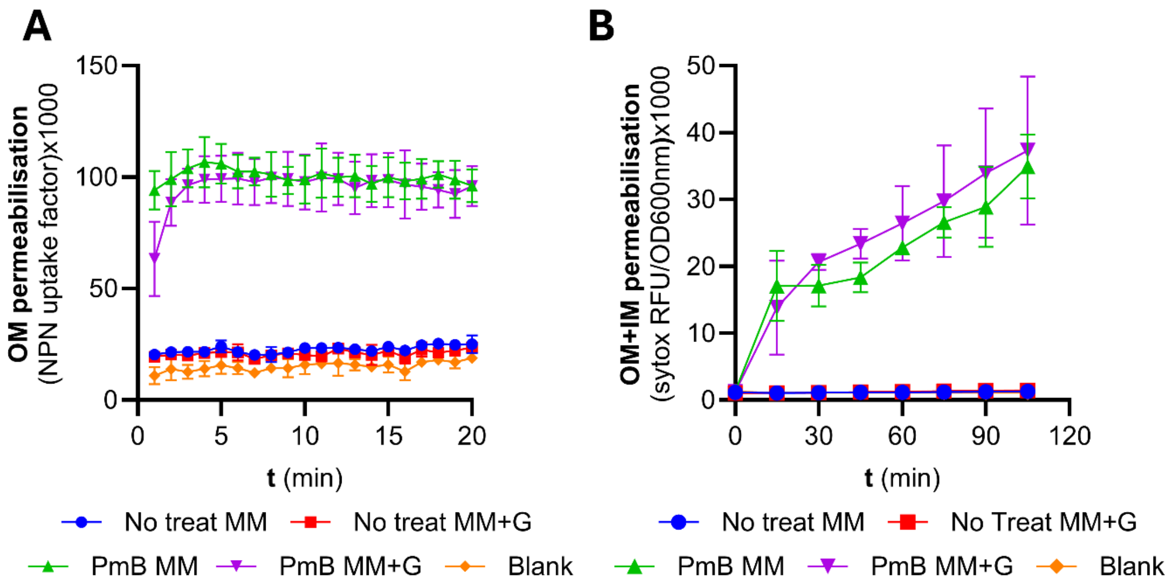

Supplementary Figure S1. Metabolic activity does not affect the ability of PmB to disrupt the OM and IM of exponential phase *E. coli.* a, OM disruption of exponential phase *E. coli* cells during the first 20 min of exposure to 4 µg ml^{-1} PmB in MM +/- glucose, as determined by uptake of the NPN fluorescent dye. **b,** OM and IM disruption of exponential phase *E. coli* exposed to 4 µg ml^{-1} PmB in MM +/- glucose, as determined by uptake of the fluorescent dye SYTOX green. The blank value refers to the relevant fluorophore in medium without bacteria. All experiments were replicated in n=3 independent assays. Error bars show the standard deviation of the mean. No significant differences were observed between exponential phase *E. coli* in MM +/- glucose for either NPN or SYTOX uptake.

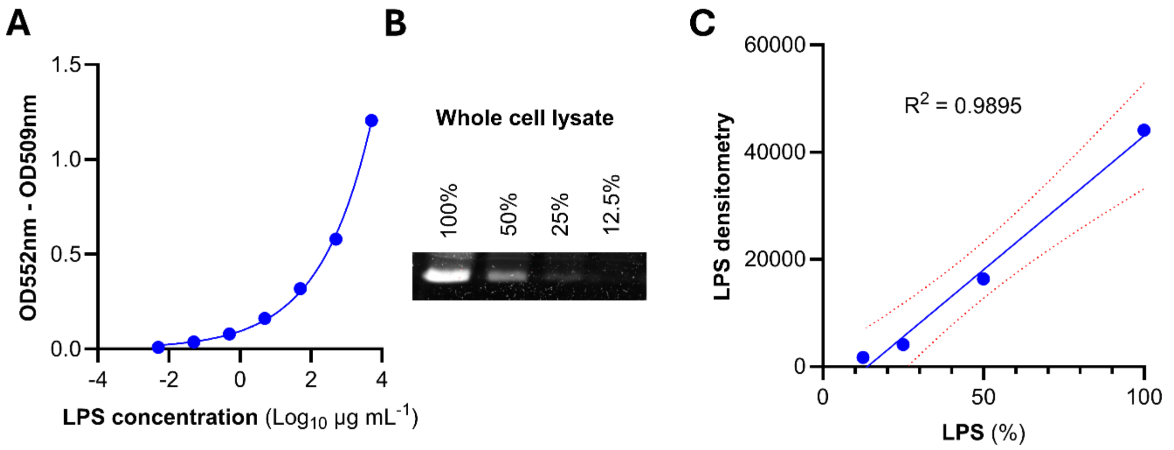

Supplementary Figure S2. The detection limit for Kdo analysis and Pro-Q™ Emerald 300 Lipopolysaccharide Gel Stain Kit. a, Kdo quantification was performed by acid hydrolysis, followed by reacting with sodium arsenite and thiobarbituric acid, yielding a coloured product. A standard curve plotting a 1:10 serial dilution series of purified rough LPS against Kdo extinction coefficient (OD552nm – OD509nm) was used to interpolate released LPS in the supernatant of stationary phase *E. coli* exposed, or not, to 4 µg ml^{-1} PmB in MM +/- glucose for 15 mins. Sigmoidal 4PL regression analysis was performed using prism version 10.4.1. **b,** The detection limit of the Pro-Q™ Emerald 300 LPS gel stain kit was determined by performing a 1:2 dilution series of stationary phase *E. coli* cells incubated in MM + glucose for 15 min. **c,** Following densitometric analysis of this dilution series, a standard curve plotting whole cell lysate LPS % against densitometry was produced. Simple linear regression was performed using prism version 10.4.1, where the blue line represents the line of best fit and the dotted red lines the 95% confidence interval.

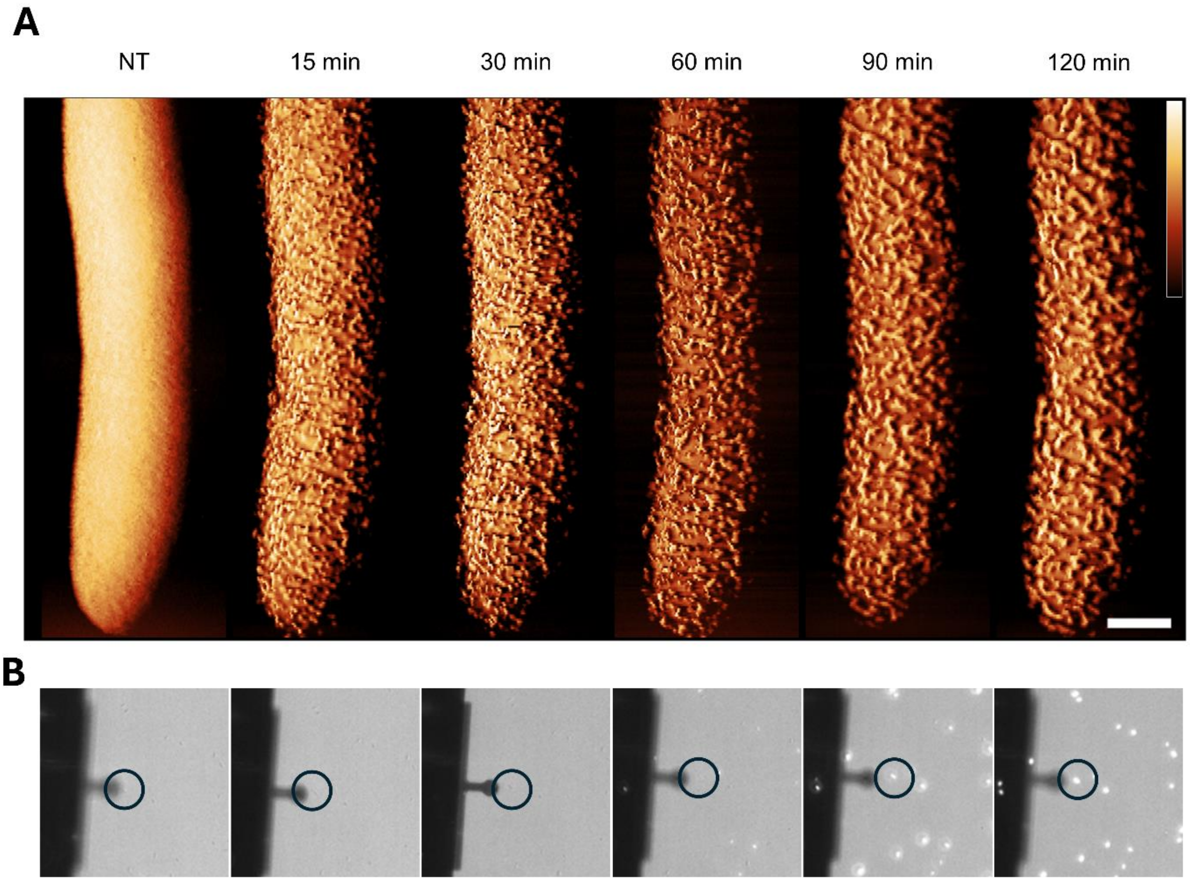

Supplementary Figure S3. a, AFM phase images showing stationary phase *E. coli* cells exposed to 2.5 μg ml^{-1} PmB in MM + G, shown as a function of time. Scalebar: 250 nm. Colour scale: 9 deg. **b,** Combined brightfield and fluorescence (SYTOX) images of the AFM scan region for the experiments in **a** (the circled cell is the one chosen for the image sequences in **a**).

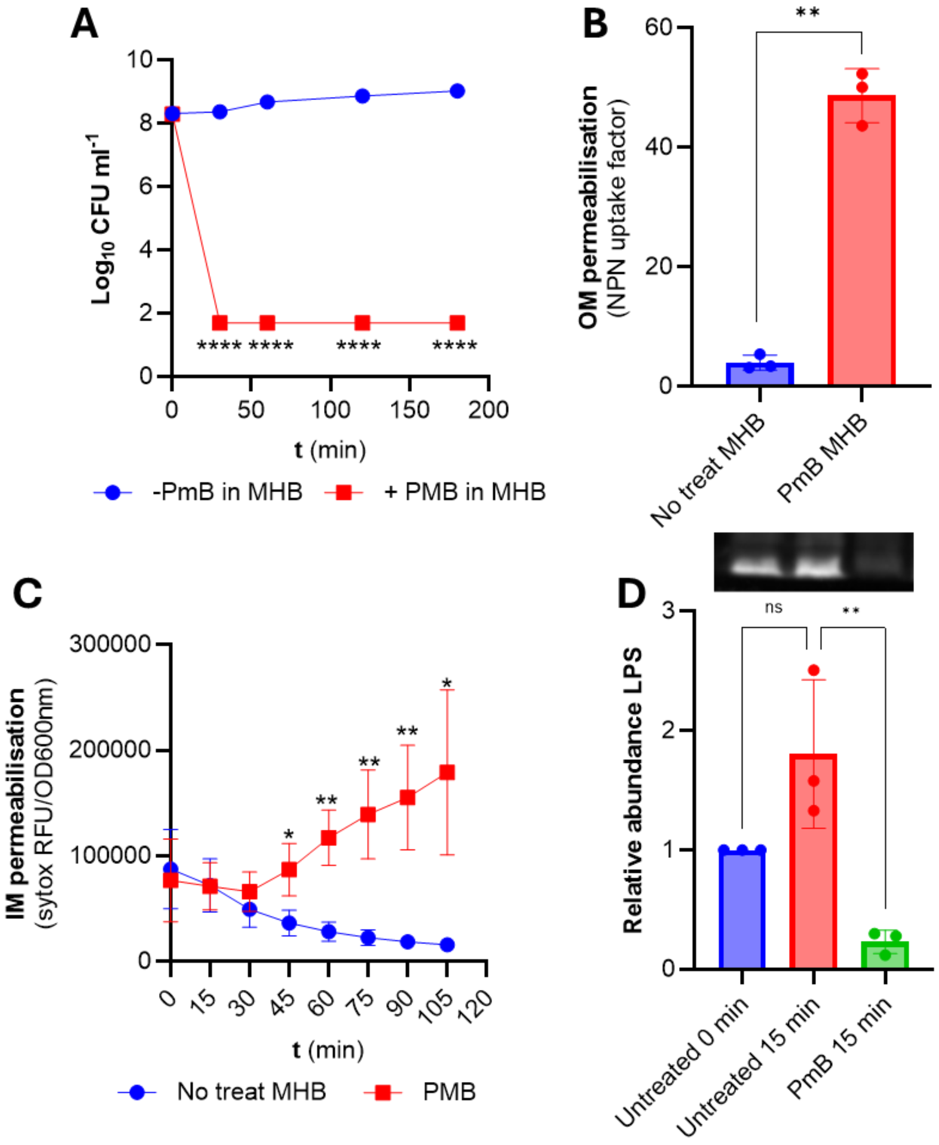

Supplementary Figure S4. PmB-induced LPS loss occurs in MHB. To ensure that LPS loss was not an artefact of performing our assays in MM or MM+G, experimentation was repeated in MHB. **a,** Survival of exponential phase *E. coli* exposed, or not, to 4 µg ml^{-1} PmB or inactive PmB, as determined by CFU counts. **b,** OM disruption of exponential phase *E. coli* cells during the first 20 min of exposure to 4 µg ml^{-1} PmB in MHB, as determined by uptake of the NPN fluorescent dye. **c,** OM and IM disruption of exponential phase *E. coli* exposed to 4 µg ml^{-1} PmB in MHB, as determined by uptake of the fluorescent dye SYTOX green. **d,** Total LPS levels of exponential phase *E. coli* exposed to 4 µg ml^{-1} PmB in MHB for 15 mins. The graph shows the quantification of LPS levels from densitometric analysis using Fiji. For all experiments n=3 independent assays, error bars show the standard deviation of the mean. Significant differences were determined by one- (**b,d**) or two-way **(a, c,)** repeated measures ANOVA. P= *<0.05, **<0.01, ***<0.001, ****<0.0001, ns = not significant.

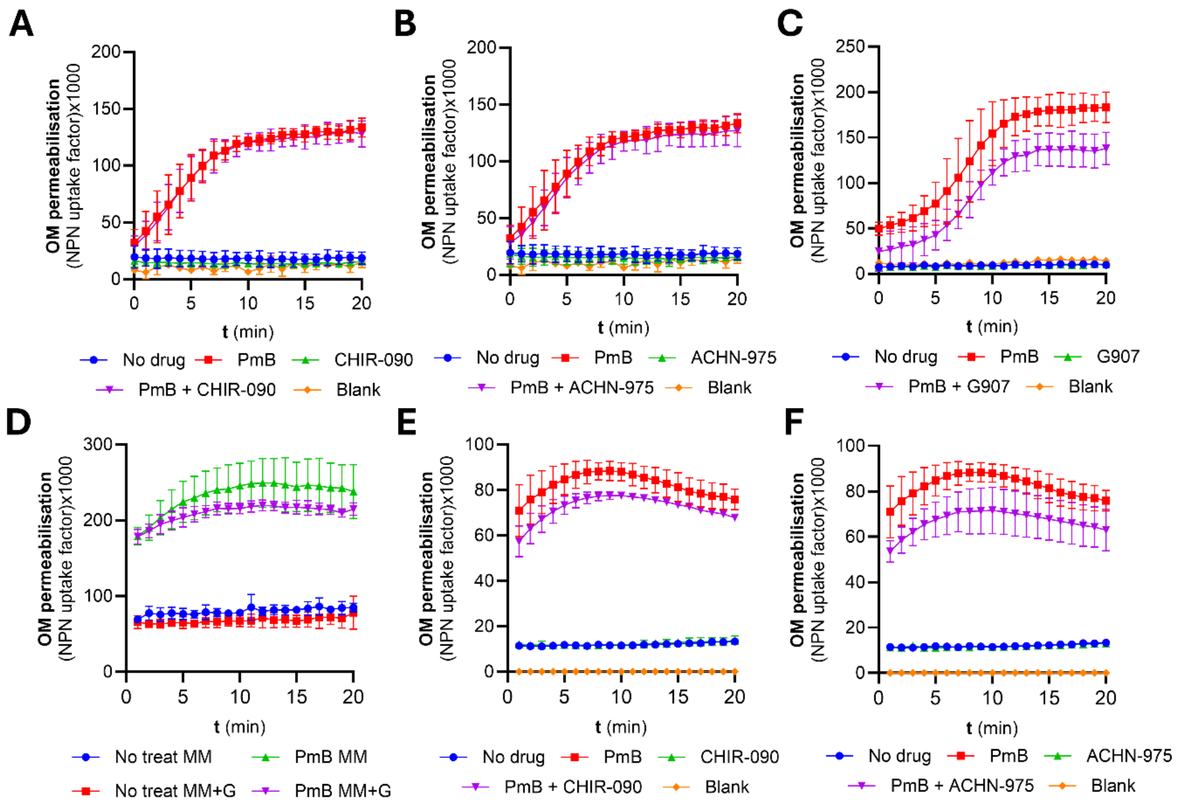

Supplementary Figure S5. Blocking LPS synthesis or transport does not affect OM permeabilisation. a, b, c, OM disruption of stationary phase *E. coli* cells during the first 20 min of exposure to 4 µg ml^{-1} PmB in MM+G with or without 1X MIC of LpxC inhibitors CHIR-090 (**a**), ACHN-975 (**b**), or the MsbA inhibitor G907 (**c**). **d,** OM disruption of stationary phase *P. aeruginosa* cells during the first 20 min of exposure with and without 4 µg ml^{-1} PmB in MM+G or MM. (**e, f**) OM disruption of stationary phase *P. aeruginosa* cells during the first 20 min of exposure to 4 µg ml^{-1} PmB in MM+G with or without 1X MIC of LpxC inhibitors CHIR-090 (**e**), ACHN-975 (**f**). All experiments were replicated in n=3 independent assays. Error bars show the standard deviation of the mean. The blank value refers to the relevant fluorophore in medium without bacteria.

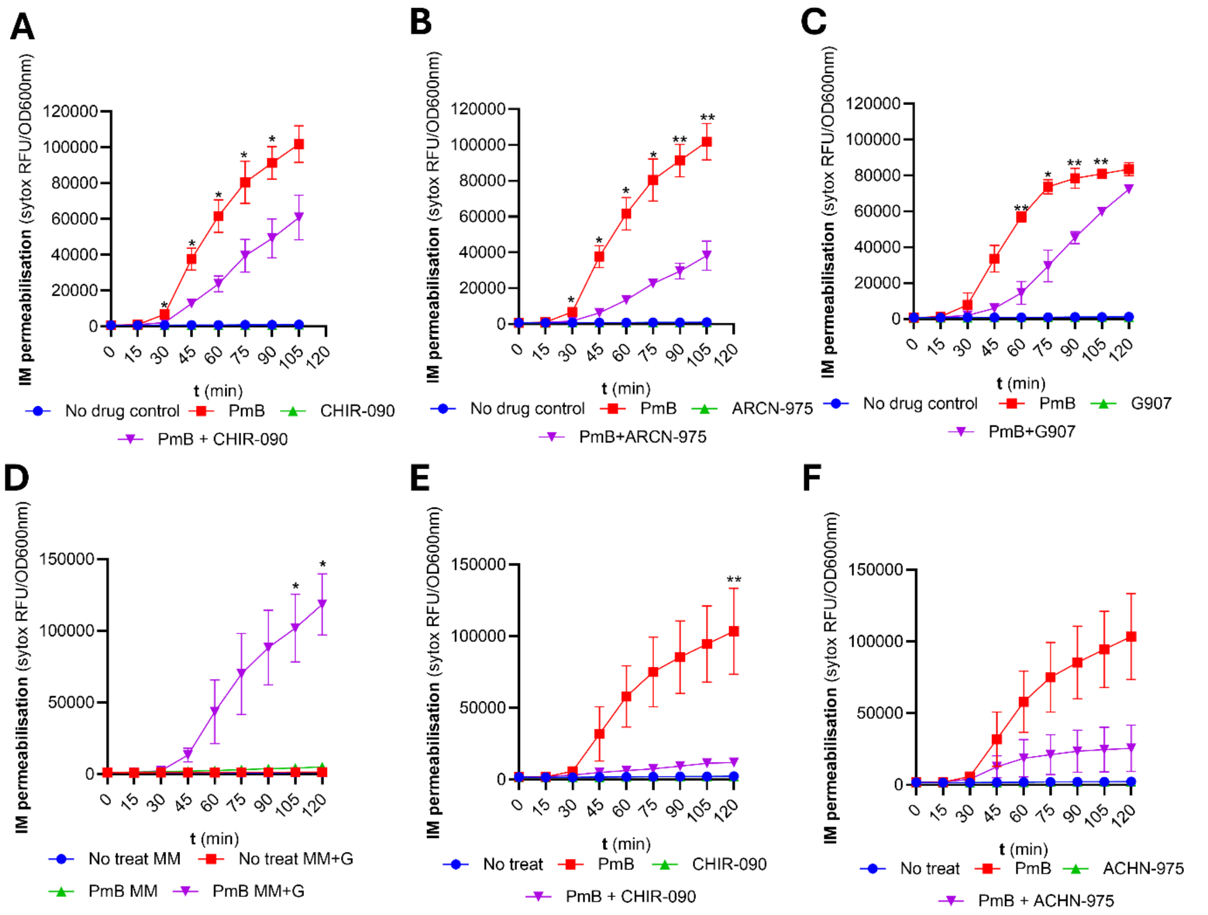

Supplementary Figure S6. Blocking LPS synthesis and transport reduces IM permeabilisation. a, b, c, OM and IM disruption of stationary phase *E. coli* exposed to 4 µg ml^{-1} PmB in MM +G, with or without 1X MIC of LpxC inhibitors CHIR-090 (**a**), ACHN-975 (**b**), or the MsbA inhibitor G907 (**c**), as determined by uptake of the fluorescent dye SYTOX green. **d,** OM and IM disruption of stationary phase *P. aeruginosa* exposed to 4 µg ml^{-1} PmB in MM +/-G. (**e, f**) OM and IM disruption of stationary phase *P. aeruginosa* exposed to 4 µg ml^{-1} PmB in MM +/-G, with or without 1X MIC of LpxC inhibitors CHIR-090 (**e**,), or ACHN-975 (**f**,). All experiments were replicated in n=3 independent assays. Error bars show the standard deviation of the mean. Significant differences were determined by two-way repeated measures ANOVA between PmB and PmB + inhibitor (**a**, **b**, **c**, **d**, **e**, **f**) or between PmB MM+G and PmB MM (**d**). P= *<0.05, **<0.01, ns=not significant.

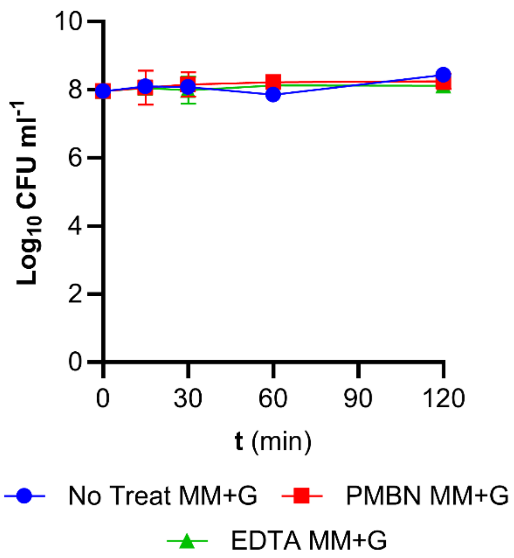

Supplementary Figure S7. PmBN and EDTA do not have bactericidal activity. Survival of *E. coli* exposed, or not, to 4 µg ml^{-1} PmBN or 10 mM EDTA in MM+G. All experiments were replicated in n=3 independent assays. Error bars show the standard deviation of the mean.

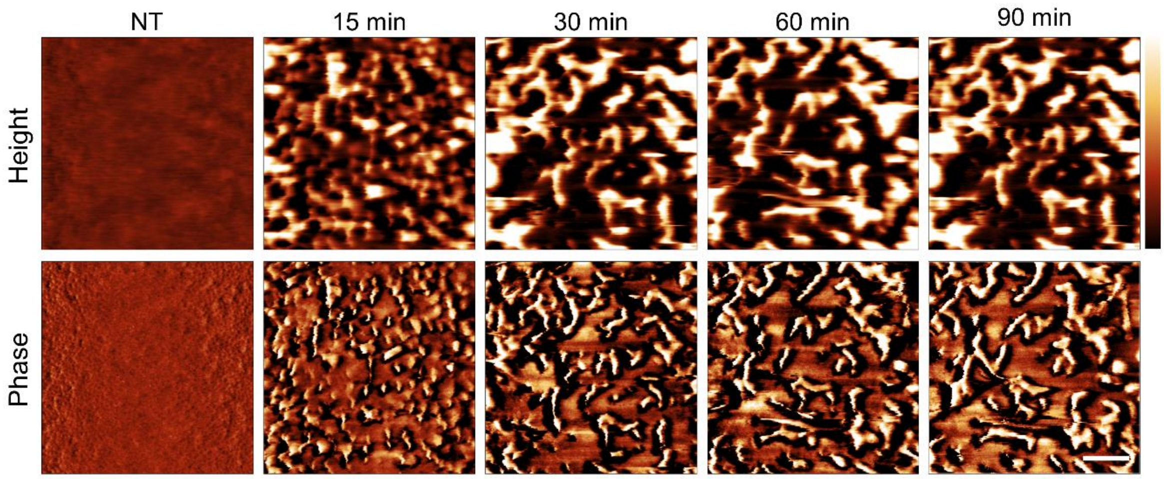

Supplementary Figure S8. In MM without glucose, co-treatment of stationary phase *E. coli* with EDTA and PmB causes clearly noticeable membrane roughening, resembling the effects observed for PmB in MM + glucose. AFM high magnification scans of the bacteria in Fig. 4H showing stationary phase *E. coli* MG1655 exposed to 10 mM EDTA and 2.5 µg ml^{-1} PmB in M9 salts followed through the time course. Scalebar: 100nm; colour bar: 20 nm (height) and 1.5 deg (phase).

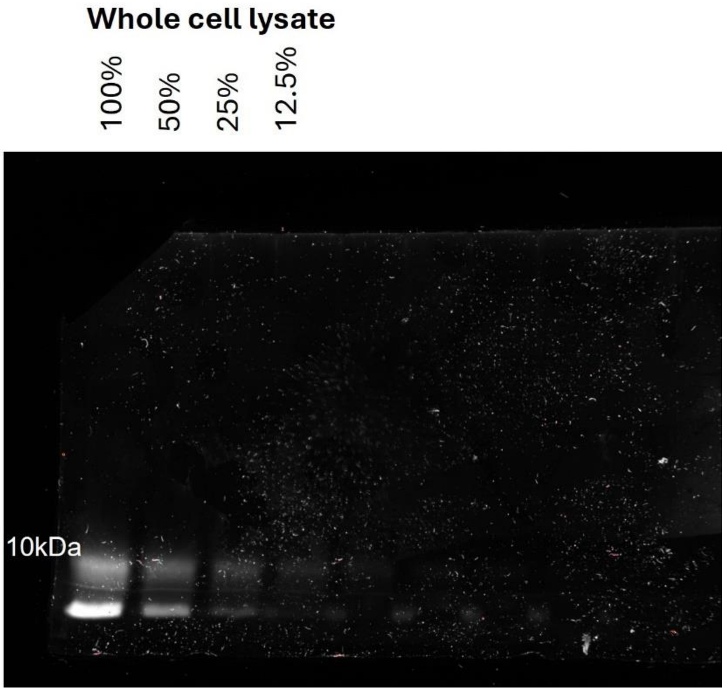

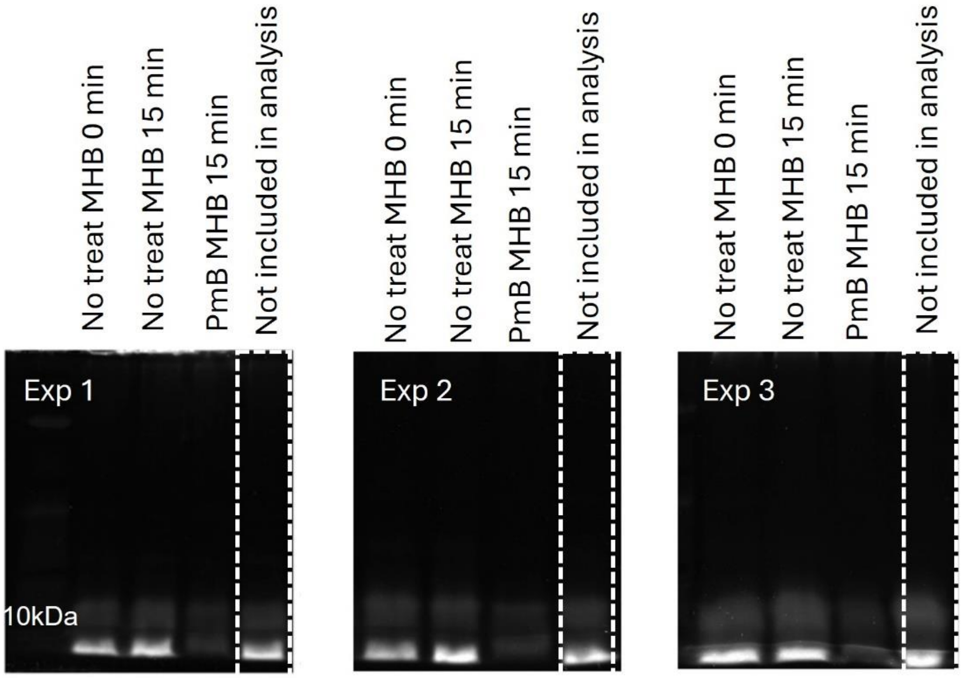

Uncropped gels for Supplementary Figure 2B (top) and Supplementary Figure 4D (bottom)
