# Supplementary figures and images for: Polymyxin B lethality requires energy-dependent outer membrane disruption
Source: Nat Microbiol. 2025 Sep 29;10(11):2919–33. doi: 10.1038/s41564-025-02133-1 (PMC12578643; doi:10.1038/s41564-025-02133-1)

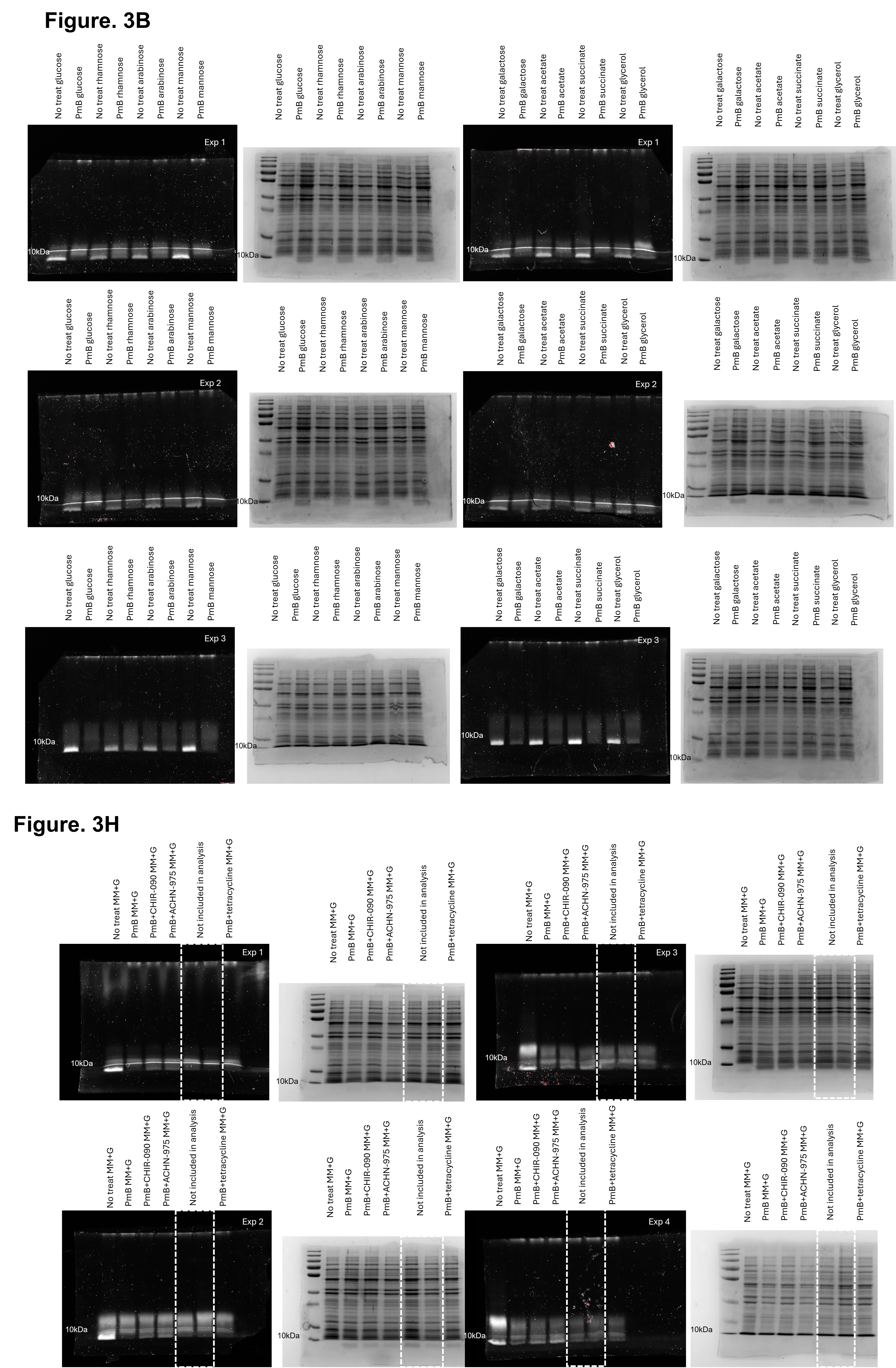

Supplement: Supplementary file 13 — Unprocessed gels. [file 41564_2025_2133_MOESM13_ESM.jpg]

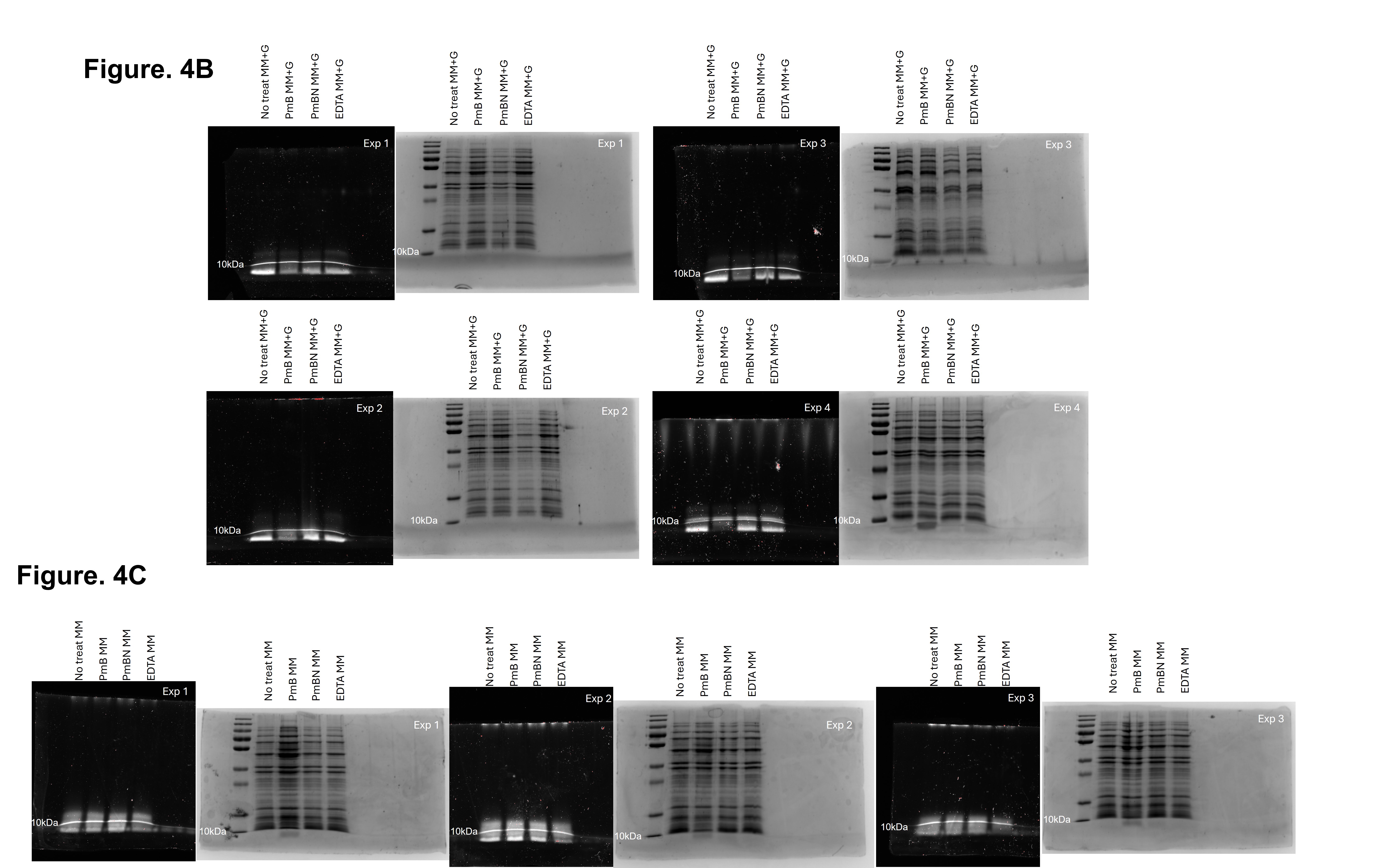

Supplement: Supplementary file 15 — Unprocessed gels. [file 41564_2025_2133_MOESM15_ESM.jpg]

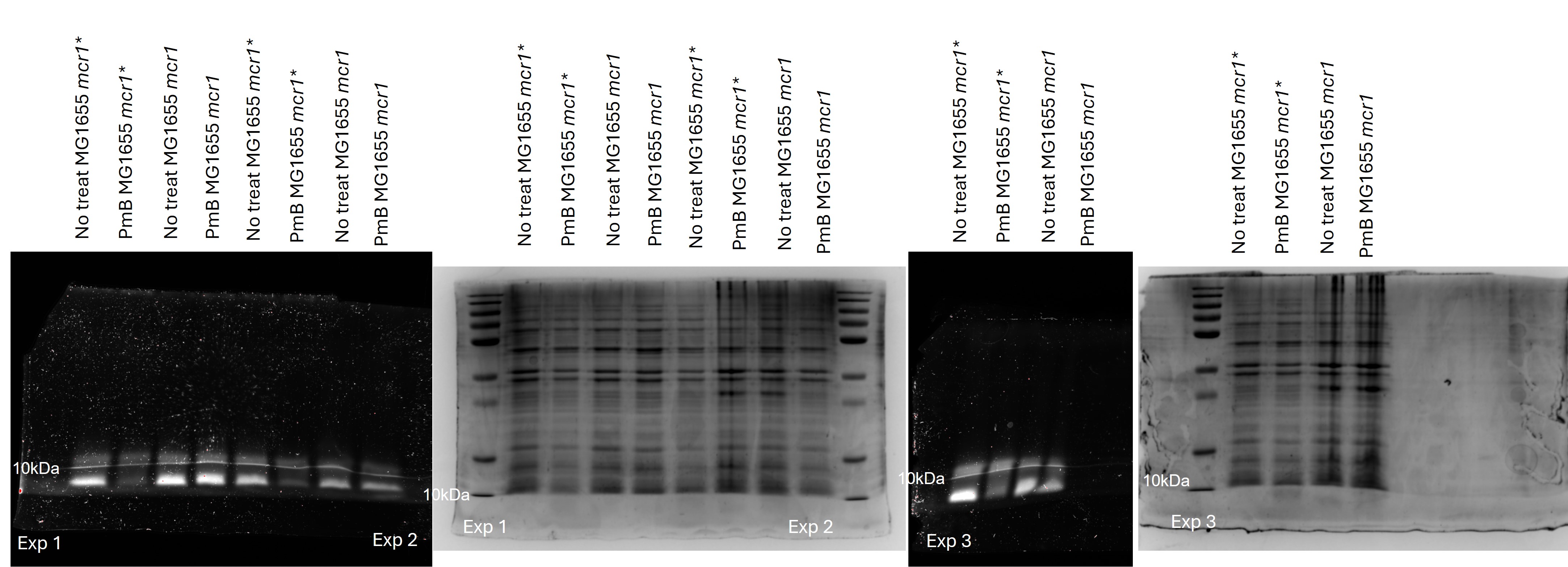

Supplement: Supplementary file 17 — Unprocessed gels. [file 41564_2025_2133_MOESM17_ESM.jpg]

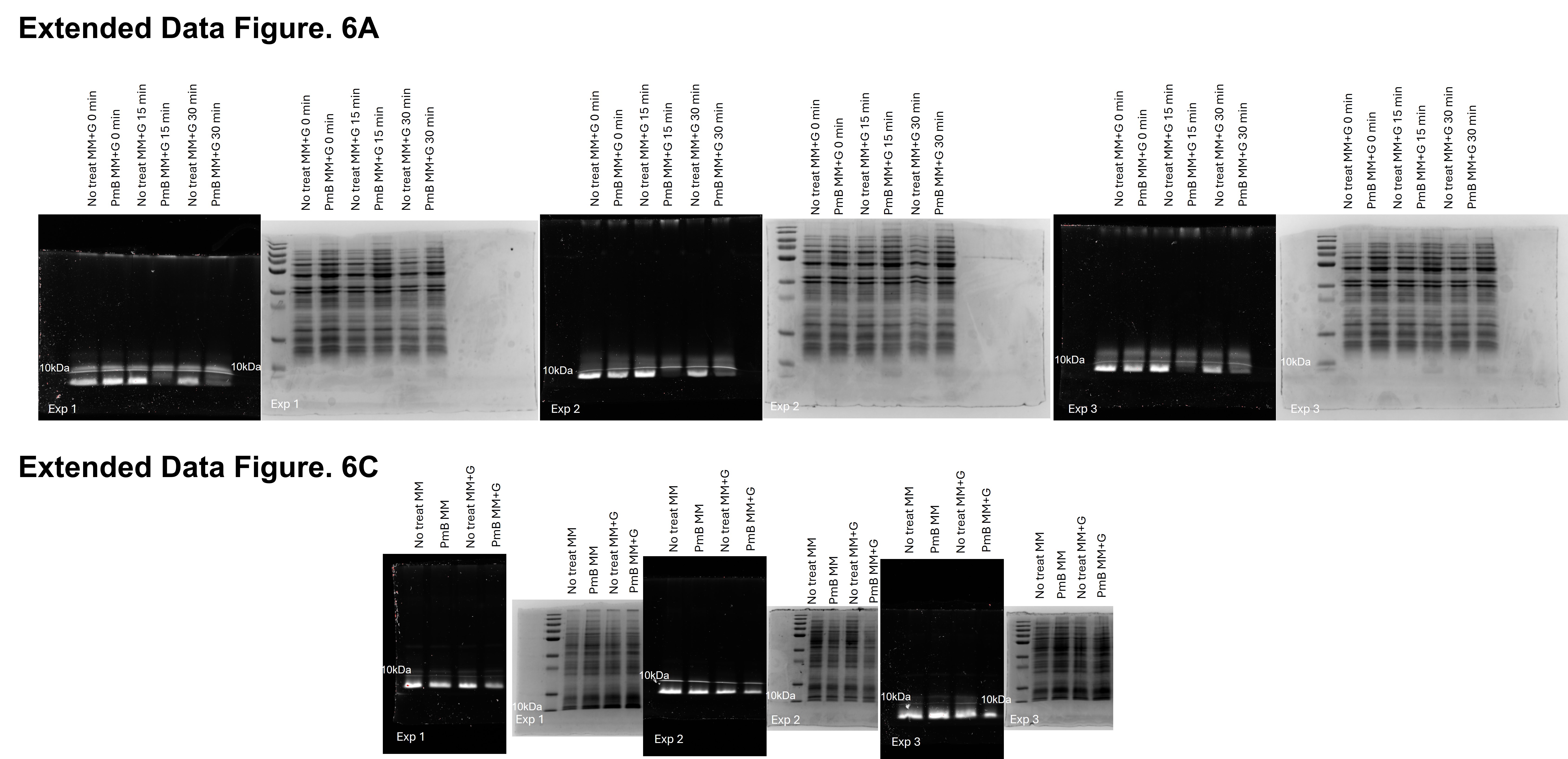

Supplement: Supplementary file 22 — Unprocessed gels for Extended Data Fig. 6a,c. [file 41564_2025_2133_MOESM22_ESM.jpg]

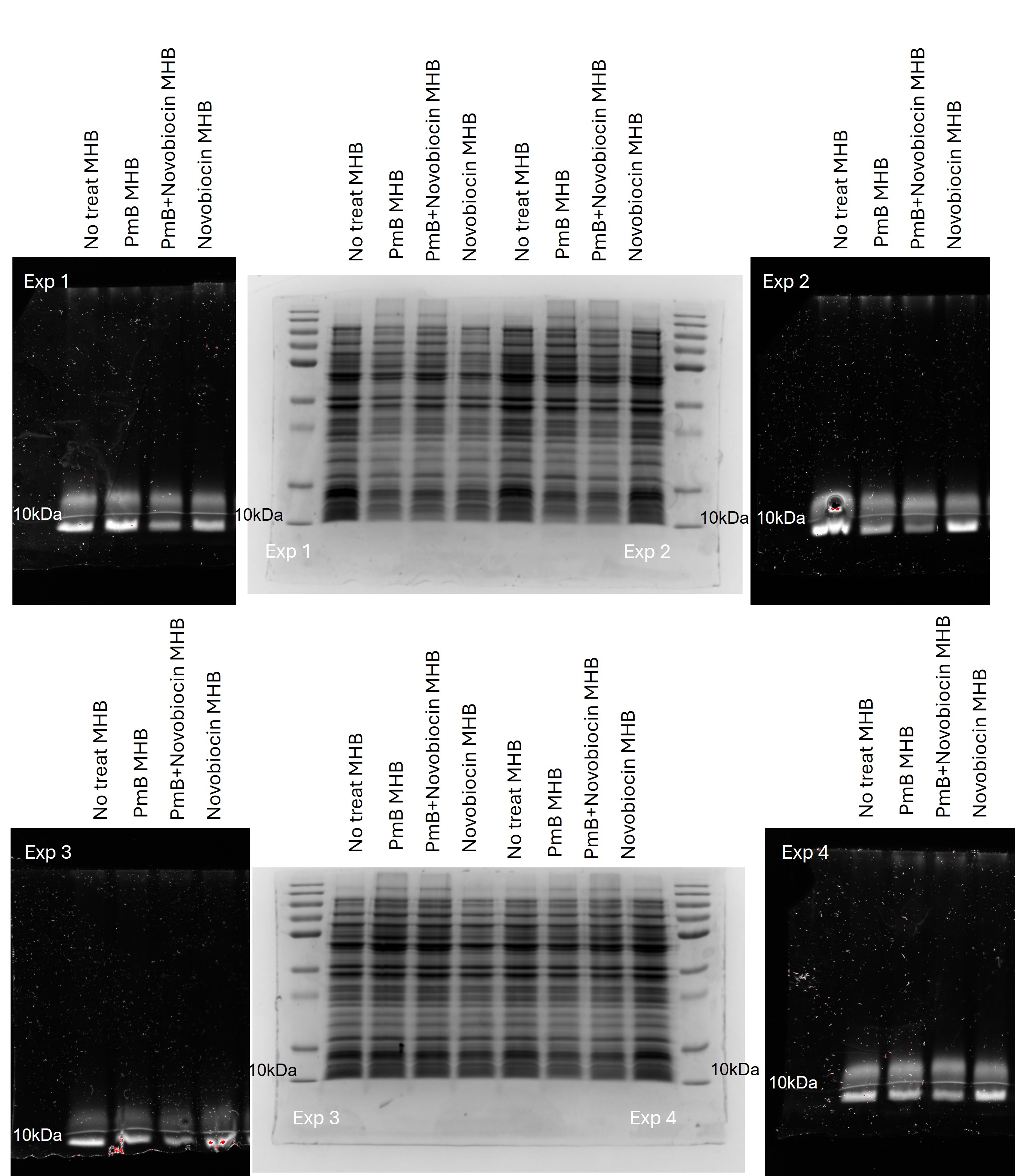

Supplement: Supplementary file 26 — Unprocessed gels for Extended Data Fig. 9g. [file 41564_2025_2133_MOESM26_ESM.jpg]
